# Highly Controllable Synthesis and DFT Calculations of Double/Triple-Halide CsPbX_3_ (X = Cl, Br, I) Perovskite Quantum Dots: Application to Light-Emitting Diodes

**DOI:** 10.3390/nano9020172

**Published:** 2019-01-30

**Authors:** Xinyue Lao, Xiyu Li, Hans Ågren, Guanying Chen

**Affiliations:** 1 School of Chemistry and Chemical Engineering, Harbin Institute of Technology, Harbin 150090, China; lao_xinyue@163.com (X.L.); hagren@kth.se (H.Å.); 2 Department of Theoretical Chemistry and Biology, Royal Institute of Technology, S-106 91 Stockholm, Sweden; xiyul@kth.se

**Keywords:** perovskite quantum dots, light-emitting diode, density functional theory, band gap

## Abstract

Although all-inorganic CsPbX_3_ (X = Cl, Br, I) perovskite quantum dots (PQDs) have evoked exciting new opportunities for optoelectronic applications due to their remarkable optical properties, their emission color tunability has not been investigated to any appreciable extent. In this work, double/triple CsPbX_3_ perovskite quantum dots with precise ratios of Cl/Br or Br/I are synthesized and their luminescence (410–700 nm) is explored. A group of down-converted CsPbX_3_ (X = Cl, Br, I) perovskite quantum dot light-emitting diode (LED) devices were constructed to demonstrate the potential use of such double/triple-halide CsPbX_3_ perovskite quantum dots with full-spectrum luminescence. Based on density functional theory, we theoretically explored the properties of CsPbX_3_ with double/triple anion atoms. The calculated band gaps provided strong support that the full-spectrum luminescence (410–700 nm) of double/triple CsPbX_3_ can be realized with the change of the mixed-halide ratios, and hence that such PQDs are of potential use in optoelectronic devices.

## 1. Introduction

During the past two decades, colloidal semiconductor quantum dots (QDs) have attracted a great deal of attention due to their unique quantum-confined optical and electrical properties [1,2]—namely, that the discrete electron energy levels near the Fermi energy level split from the continuum when the particle size is smaller than or comparable to the Bohr exciton radius. The most common quantum dot system contains cadmium-based chalcogenides [3,4,5], which exhibit efficient size-dependent luminescence properties after proper surface passivation (i.e., shelling). However, the demanding synthetic chemistry for the stringent size control, the low tolerance of luminescence properties to defects, and the containment of poisonous heavy metal elements (e.g., cadmium) raise serious concerns for their practical photonic and biophotonic applications. To overcome these problems, new types of quantum dots have been investigated. Among them, much effort has recently been devoted to perovskite quantum dots, including inorgano-organometallic halide perovskites CH_3_NH_3_PbX_3_ [6,7,8], and all-inorganic halide perovskites CsPbX_3_ (X = Cl, Br, I). Compared with the organic–inorganic hybrid perovskite quantum dots, all-inorganic CsPbX_3_ (X = Cl, Br or I) perovskite quantum dots (hereafter denoted as CsPbX_3_ PQDs) show better performance, including ultra-high photoluminescence quantum yields, narrow emission bandwidth, large extinction coefficient, high tolerance to crystal defects, and good stability against moisture and oxygen [9,10]. Moreover, the fact that the emission wavelengths of CsPbX_3_ PQDs can be tuned precisely by their composition instead of their size offers a significant advantage over traditional QDs, avoiding the need for complicated and demanding chemistry to produce a narrow band emission. Indeed, CsPbX_3_ PQDs with tunable and bright photoluminescent (PL) properties have been synthesized with different methods such as hot injection [11], anion exchange [12], ultrasonication [13], solvothermal [14], and microwave-assisted [15] methods, and different shapes have been designed [14] to obtain better photoelectric performance. Owing to their excellent photoelectric performance, CsPbX_3_ PQDs have great application potential in fields such as solar cells [16,17], light-emitting diodes (LEDs) [18,19], lasers [20], and photodetectors [21]. Although significant progress has been made in the synthesis and application of CsPbX_3_ PQDs, they still need to be explored by providing a comprehensive composition-defined luminescence and by theoretical calculation of structure and properties like band gaps and formation energies.

Herein, we report the synthesis of double/triple-halide CsPbX_3_ (X = Cl, Br, I) PQDs with precise ratios of Cl/Br or Br/I, using the hot-injection method. A series of light-emitting diodes covering the entire visible spectrum were fabricated and investigated. Furthermore, we applied a comprehensive approach to calculate the band gap and formation energy of the synthesized double/triple-halide CsPbX_3_ (X = Cl, Br, I) PQDs, namely, the projector-augmented plane wave (PAW) method [22] within the framework of density functional theory (DFT) implemented in the Vienna Ab-initio Simulation Package (VASP) [23]. Calculated data are presented and discussed in comparison with the experimental results.

## 2. Results and Discussion

**Figure 1:**
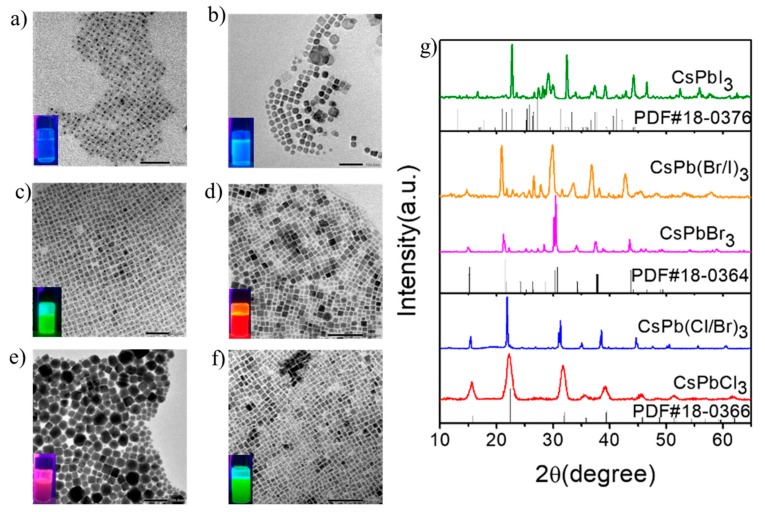
TEM images of (**a**) CsPbCl_3_; (**b**) CsPb(Cl/Br)_3_; (**c**) CsPbBr_3_; (**d**) CsPb(Br/I)_3_; (**e**) CsPbI_3_; (**f**) CsPb(Cl/Br/I)_3_ (I insets are photographs of CsPbX_3_ PQDs under 365 nm UV excitation); (**g**) XRD patterns of CsPbX_3_ (X = Cl, Br, I, Cl/Br, and Br/I).

Figure 1a–f shows typical low-magnification transmission electron microscopy (TEM) images of the synthesized single- or double/triple-halide CsPbX_3_ (X = Cl, Br, or I) PQDs. As seen in these figures, most of the CsPbX_3_ (X = Cl, Br, or I) PQDs had a monodisperse cubic shape. The single CsPbCl_3_ PQDs and CsPbBr_3_ PQDs seen in Figure 1a,c showed a relatively uniform cubic shape with a particle size of about 10 nm. The cubic morphology of the CsPbI_3_ PQDs seen in Figure 1e is an exception, being stable only at high temperature. The double-halide CsPb(Cl/Br)_3_ PQDs and CsPb(Br/I)_3_ PQDs, and the triple-halide CsPb(Cl/Br/I)_3_ PQDs, seen respectively in Figure 1b,d,f, also showed cubic shape with an average edge length of 12 nm. Figure 1g presents corresponding XRD patterns of the synthesized single- or double-halide CsPbX_3_ (X = Cl, Br, or I) PQDs, which can be indexed as cubic phase CsPbCl_3_ (PDF 18-0366) and CsPbBr_3_ (PDF 18-0364), respectively.

**Figure 2:**
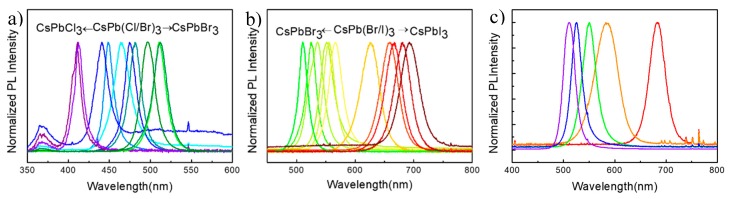
Normalized photoluminescence (PL) intensity of the synthesized double-halide CsPbX_3_ (X = Cl, Br, or I) PQDs. (**a**) Normalized PL intensity of CsPb (Cl/Br)_3_. (**b**) Normalized PL intensity of CsPb (Br/I)_3_. (**c**) Normalized PL intensity of CsPb (Cl/Br/I)_3_.

Figure 2a,b shows normalized photoluminescence (PL) spectra of the synthesized double-halide CsPbX_3_ (X = Cl, Br, or I) PQDs. With an increasing molar ratio of Br/Cl, the PL emission of the double-halide CsPb(Cl/Br)_3_ PQDs moved gradually from 410 to 515 nm. In the same way, the PL emission of the double-halide CsPb(Br/I)_3_ PQDs changed gradually from 515 to almost 692 nm. With the purpose of controlling the PL emission wavelength more accurately, a series of triple-halide CsPb(Cl/Br/I)_3_ PQDs were synthesized and studied. Specifically, the amount of added PbCl_2_ remained the same while the molar ratio of I/Br was adjusted. As shown in Figure 2c, a difference with the double-halide PQDs was that the central wavelength of the emission peak was positively proportional to the molar ratio of I/Br. A linear relation between the peak and molar ratio could be obtained by curve fitting. The results indicate that quantum dots with full-spectrum (410–700 nm) luminescence could be synthesized by all three kinds of halogen doping.

**Figure 3:**
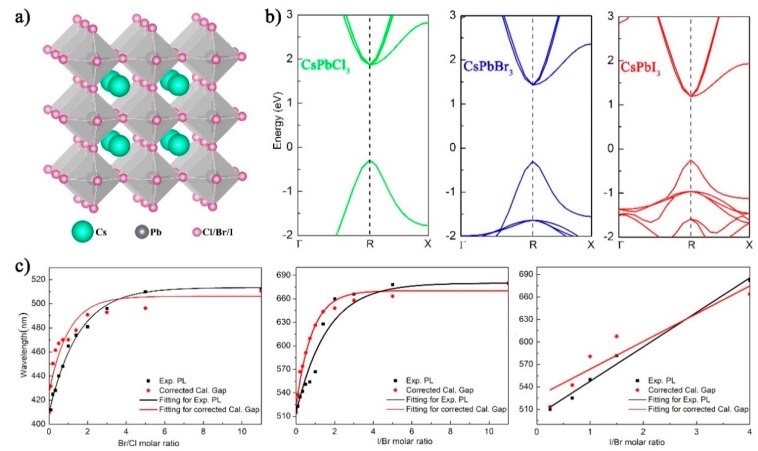
(**a**) The atom structure of halide perovskites. (**b**) Band structure for pure phase of CsPbX_3_ (X = Cl, Br, or I). (**c**) Band gaps versus Br/Cl molar ratio.

We theoretically examined the stability and electronic structures of CsPbX_3_ with double/triple anion atoms, which determined their optical performances. The DFT projector-augmented plane wave (PAW) method [23,24] was used in conjunction with the generalized gradient approximation (GGA) of the Perdew, Burke, and Ernzerhof (PBE) functional [25] for electron exchange and correlation. To investigate the properties of mixed-halide perovskites CsPbCl_3(1−x)_Br_3x_, CsPbBr_3(1−x)_I_3x_, CsPbCl_3(1−x−y)_Br_3x_I_3y_, supercells of halide perovskites containing 2 × 2 × 2 unit cells were constructed and simulated. A plane-wave cutoff of 500 eV was used. The Brillouin zone was sampled with 5 × 5 × 5 Monkhorst–Pack k-meshes, as seen in Figure 3a. All the structures were fully relaxed with a force tolerance of 0.02 eV/Å. It was reported that mixed-halide perovskites, where the structure with Cl, Br, and I atoms are distributed randomly at the halide sites, are stable [26,27]. To examine the mixed-halide perovskites further, we calculated the formation energies (ΔH) to assess their relative enthalpic stability by the subsequent formulation:

(1) $$\Delta H\left(A_{1-x-y}B_{x}C_{y}\right)=E\left(A_{1-x-y}B_{x}C_{y}\right)-\left(1-x-y\right)E\left(A\right)-xE\left(B\right)-yE\left(C\right).$$

Specifically, for one certain concentration of a mixed halide, more than five atom-structure models with different halide atom arrangements were used for simulations of the double/triple CsPbX_3_ PDQs. The model with the smallest calculated value of ΔH was considered the most stable, which was then used to simulate this particular concentration of the mixed-halide perovskite. Among these structures, relatively, the largest positive value of formation energy for the triple-halide-structure 3 (ΔH = 0.275 eV) indicates that this structure is the most unstable one (as shown in Table 1, Table 2 and Table 3). Subsequently, we performed molecular dynamics simulations to examine the structural stability of the triple-halide-3 at a room temperature of 300 K. The simulation indicated that the structure of triple-halide-structure 3 did not show obvious changes, suggesting that crystals with this structure are metastable at least. Thus, the stability of triple-halide-structure 3 with the largest calculated value of ΔH implies that the other structures of the mixed-halide perovskite with the smaller value of ΔH are more stable or actually stable. Calculating the electronic structures of the mixed-halide perovskites, it was found that the band gaps of CsPbX_3_ were underestimated compared with the experimental data of the emission peaks due to the nature of the PBE method, as seen in Figure 3b. Therefore, to properly characterize the change of trends of the band gaps, we used the formulations given in the Appendix A to correct the results, as shown in Figure 3c where the corrected band gaps then get close to the experimental values). The band gaps of the mixed-halide perovskite are a function of the mixed-halide ratio, and agreed with the experimental emission peaks. One can thus understand that the band gap of the perovskite can be tuned by changing the mixed-halide ratio, which is the fundamental reason for the realization of full-spectrum luminescence (410–700 nm) of double/triple CsPbX_3_ PQDs. In particular, all the mixed-halide perovskites have a direct band structure, which contributes to the high photoemission quantum yields for perovskites with mixed halides.

**Figure 4:**
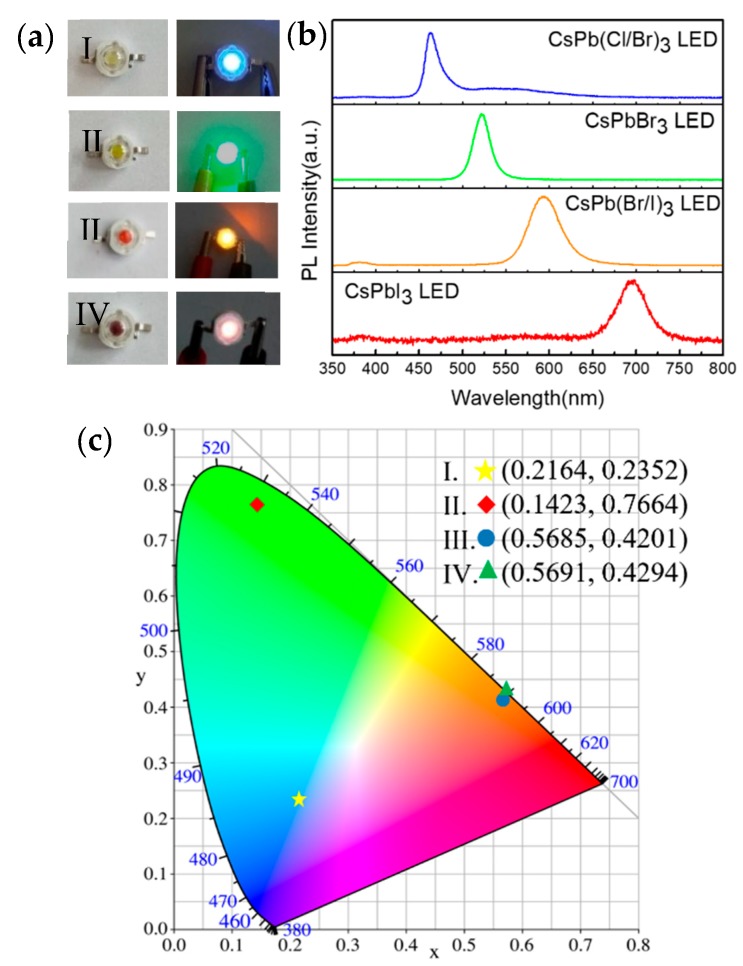
(**a**) The fabricated CsPbX_3_ perovskite quantum dots (PQDs) light-emitting diode (LED) device. (**b**) The emission spectrum of the fabricated CsPbX_3_ LED devices. (**c**) The CIE (Commission Internationale de l’Eclairage) coordinates of the fabricated PQDs LED device.

Since the wavelength of the double/triple-CsPbX_3_ (X = Cl, Br, or I) PQDs can be precisely controlled, a group of down-converted light-emitting diodes (LEDs) were constructed to demonstrate the potential use of double/triple-CsPbX_3_ (X = Cl, Br, or I) PQDs for full-spectrum luminescence. Figure 4b shows the emission spectrum of the fabricated CsPbX_3_ LED devices with emission peaks located in a diverse range. As demonstrated by Figure 4a, the fabricated CsPbX_3_ PQDs LED device emitted different bright colors when a 3 V power source was applied. The CIE (Commission Internationale de l’Eclairage) color coordinates of the fabricated PQDs LED device are shown in Figure 4c, which illustrates the different colors of the fabricated CsPbX_3_ PQDs LED devices. These results indicate that the as-prepared CsPbX_3_ PQDs are promising materials for future applications in full-spectrum display devices.

## 3. Conclusions

In summary, we synthesized all-inorganic CsPbX_3_ (X = Cl, Br, I) perovskite quantum dots with full-spectrum (410–700 nm) luminescence by means of the hot-injection method using the molar ratio of Br/Cl and I/Br to precisely control the luminescence wavelength. Based on the DFT method, we theoretically explored the properties of CsPbX_3_ with double/triple anion atoms. The calculated band gaps strongly support the variation of the emission peaks upon the mixed-halide ion ratio. The results of the calculations suggest that the change of the mixed-halide ion ratio is the fundamental reason for the realization of the full-spectrum luminescence (410–700 nm) of double/triple CsPbX_3_ materials. A series of down-converted LED devices was systematically investigated, indicating that the as-prepared CsPbX_3_ PQDs could be promising materials for future applications in full-spectrum display devices.

## 4. Experimental Section

*Chemicals and Reagents*: Cesium carbonate (Cs_2_CO_3_, 99.9%), 1-octadecene (ODE, ≥90%), oleic acid (OA, AR), oleylamine (OAm, 80–90%), lead chloride (PbCl_2_, 99.999%), lead bromide (PbBr_2_, 99.999%), and lead iodide (PbI_2_, 99.999%) were purchased from Aladdin (Shanghai, China). Hexane (≥95%) was purchased from Tianjin Chemical Works (Tianjin, China). All of the reagents were used without further purification.

*Synthesis of Cs-Oleate Precursors*: First, Cs_2_CO_3_ (0.816 g, 2.5 mmol), OA (2.5 mL), and ODE (40 mL) were added to a 250 mL three-neck flask and stirred at 125 °C under flowing nitrogen. After a 45-min reaction, the mixture became a clear solution and the Cs-oleate precursors were obtained. The Cs-oleate should be preheated to 100 °C before injection to maintain accuracy because Cs-oleate precipitates out of ODE at room temperature.

*Synthesis of Single- or Double/Triple-Halide CsPbX3 (X = Cl, Br, or I) PQDs*: Colloidal CsPbX_3_ PQDs were synthesized following Protesescu’s procedure [6]. First, 0.6 mmol PbX_2_ (X = Cl, Br, or I) mixed by a certain molar ratio and 14 mL ODE was added to a 250 mL three-neck flask. Then, the mixture was stirred and heated to 125 °C under flowing nitrogen. After 45 min, OA (2 mL) and OAm (2 mL) were injected into the reaction system to completely solubilize PbX_2_ and the temperature was raised to 160 °C in the meantime. When a clear solution was obtained, the Cs-oleate precursor (3 mL), preheated to 100 °C, was quickly injected. After 10 s, the reaction system was cooled down by an ice-water bath to terminate the reaction. In order to get pure products, the turbid liquid was centrifuged at 6000 rpm for 5 min. After centrifugation, the supernatant was discarded and the precipitate at the bottom of the centrifuge tube was dispersed in 2 mL hexane. The centrifugation procedure was repeated at 10,000 rpm for 10 min. The final product was redispersed in 2 mL hexane and stored following purification.

*PQDs LED Device Fabrication*: The main agent A was mixed with the 10 μL n-hexane solution containing the PQDs and stirred thoroughly before adding the curing agent B. After adding the curing agent, the stirring time usually took 5 min. The PQDs powder and the agent were completely mixed, and the bubbles were removed by low-frequency ultrasound for subsequent use. The agent containing the PQDs was properly stirred and placed in the holder cup of the mold. The mold was put under UV light for 5 min to solidify the agent.

*Characterization*: The size and morphology of CsPbX_3_ PQDs were characterized by transmission electron microscopy (TEM) using a JEOL JEM-2010 microscope (JEOL Ltd., Tokyo, Japan) at an acceleration voltage of 200 kV. The PL spectra were measured by a spectrometer (Ocean Optics FLAME-S-VIS-NIR) under excitation at 365 nm using a UV lamp. X-ray diffraction (XRD) patterns were obtained using a D8 Focus X-ray diffractometer.

## Figures and Tables

**Table 1:** The calculated formation energies of the mixed-halide perovskite CsPb(Cl_1−x_Br_x_)_3_.

	Percentage of Cl	Percentage of Br	Cl:Br Molar Ratio	ΔH (eV)
1	8.3%	91.7%	1:11	0.041
2	16.7%	83.3%	2:10	0.035
3	25%	75%	3:9	0.142
4	33.3%	66.7%	4:8	0.071
5	41.7%	58.3%	5:7	0.092
6	50%	50%	6:6	0.186
7	58.3%	41.7%	7:5	0.108
8	66.7%	33.3%	8:4	0.050
9	75%	25%	9:3	0.067
10	83.3%	16.7%	10:2	0.067
11	91.7%	8.3%	11:1	0.071

**Table 2:** The calculated formation energies of the mixed-halide perovskite CsPb(Br_1−y_I_y_)_3_.

	Percentage of Br	Percentage of I	Br:I Molar Ratio	ΔH (eV)
1	8.3%	91.7%	1:11	0.041
2	16.7%	83.3%	2:10	0.035
3	25%	75%	3:9	0.142
4	33.3%	66.7%	4:8	0.071
5	41.7%	58.3%	5:7	0.092
6	50%	50%	6:6	0.186
7	58.3%	41.7%	7:5	0.108
8	66.7%	33.3%	8:4	0.050
9	75%	25%	9:3	0.067
10	83.3%	16.7%	10:2	0.067
11	91.7%	8.3%	11:1	0.071

**Table 3:** The calculated formation energies of the mixed-halide perovskite CsPb(Cl_1−x−y_Br_x_I_y_)_3_.

	Cl:Br:I Molar Ratio	ΔH (eV)
1	2:8:2	0.129
2	2:6:4	0.185
**3**	**2:5:5**	**0.275**
4	2:4:6	0.252
5	2:2:8	0.214

## Appendix A

To correctly characterize the change of trends of the band gaps, we used the following formulations to correct the calculated results:

(A1) $$\Delta \lambda \left(x\right)=x*\Delta \lambda_{1}+\left(1-x\right)*\Delta \lambda_{2}+m*x*\left(1-x\right),\left(m=40\;\mathrm{nm}\right)\;\Delta \lambda_{1}:\;\lambda_{cal}-\lambda_{exp}\;\mathrm{For}\;\mathrm{pure}\;{\mathrm{CsPbBr}}_{3}\;\Delta \lambda_{2}:\;\lambda_{cal}-\lambda_{exp}\;\mathrm{For}\;\mathrm{pure}\;{\mathrm{CsPbCl}}_{3}\;x:\;\mathrm{The}\;\mathrm{Br-doping}\;\mathrm{concentration}$$

(A2) $$\Delta \lambda \left(x\right)=x*\Delta \lambda_{1}+\left(1-x\right)*\Delta \lambda_{2}+m*x*\left(1-x\right),\left(m=30\;\mathrm{nm}\right)\;\Delta \lambda_{1}:\;\lambda_{cal}-\lambda_{exp}\;\mathrm{For}\;\mathrm{pure}\;{\mathrm{CsPbI}}_{3}\;\Delta \lambda_{2}:\;\lambda_{cal}-\lambda_{exp}\;\;\mathrm{For}\;\mathrm{pure}\;{\mathrm{CsPbBr}}_{3}\;x:\mathrm{The}\;\mathrm{I-doping}\;\mathrm{concentration}$$

(A3) $$\Delta \lambda \left(x\right)=\frac{2}{12}*\Delta \lambda_{Cl}+x*\Delta \lambda_{1}+\left(\frac{10}{12}-x\right)*\Delta \lambda_{2}+m*\frac{2}{12}*x*\left(\frac{10}{12}-x\right),\left(m=100\;\mathrm{nm}\right)\;\Delta \lambda_{Cl}:\;\lambda_{cal}-\lambda_{exp}\;\mathrm{For}\;\mathrm{pure}\;{\mathrm{CsPbCl}}_{3}\;\Delta \lambda_{1}:\;\lambda_{cal}-\lambda_{exp}\;\mathrm{For}\;\mathrm{pure}\;{\mathrm{CsPbI}}_{3}\;\Delta \lambda_{2}:\;\lambda_{cal}-\lambda_{exp}\;\mathrm{For}\;\mathrm{pure}\;{\mathrm{CsPbBr}}_{3}\;x:\;\mathrm{The}\;\mathrm{I-doping}\;\mathrm{concentration}$$
